# Myosin VI Regulates Actin Structure Specialization through Conserved Cargo-Binding Domain Sites

**DOI:** 10.1371/journal.pone.0022755

**Published:** 2011-08-11

**Authors:** Mamiko Isaji, Marta Lenartowska, Tatsuhiko Noguchi, Deborah J. Frank, Kathryn G. Miller

**Affiliations:** 1 Department of Biology, Washington University in St. Louis, St. Louis, Missouri, United States of America; 2 Faculty of Biology and Earth Sciences, Institute of General and Molecular Biology, Laboratory of Developmental Biology, Nicolaus Copernicus University, Torun, Poland; 3 Laboratory for Morphogenetic Signaling, Center for Developmental Biology, RIKEN Kobe, Kobe, Japan; Université de Genève, Switzerland

## Abstract

Actin structures are often stable, remaining unchanged in organization for the lifetime of a differentiated cell. Little is known about stable actin structure formation, organization, or maintenance. During Drosophila spermatid individualization, long-lived actin cones mediate cellular remodeling. Myosin VI is necessary for building the dense meshwork at the cones' fronts. We test several ideas for myosin VI's mechanism of action using domain deletions or site-specific mutations of myosin VI. The head (motor) and globular tail (cargo-binding) domains were both needed for localization at the cone front and dense meshwork formation. Several conserved partner-binding sites in the globular tail previously identified in vertebrate myosin VI were critical for function in cones. Localization and promotion of proper actin organization were separable properties of myosin VI. A vertebrate myosin VI was able to localize and function, indicating that functional properties are conserved. Our data eliminate several models for myosin VI's mechanism of action and suggest its role is controlling organization and action of actin assembly regulators through interactions at conserved sites. The Drosophila orthologues of interaction partners previously identified for vertebrate myosin VI are likely not required, indicating novel partners mediate this effect. These data demonstrate that generating an organized and functional actin structure in this cell requires multiple activities coordinated by myosin VI.

## Introduction

Studies of motile cells have revealed a complex network of proteins that orchestrates the assembly and disassembly of actin structures that mediate movement. Cell shape constantly changes and the actin structures that are important for cell shape and movement rapidly and constantly reorganize. In contrast, many differentiated cells that make up multicellular organisms are not motile. The actin cytoskeleton plays important roles in the processes that occur during development as cells become specialized and in physiological functions of the many different cell types in multicellular organisms. For example, actin filaments are important for the cell-cell and cell-substrate contacts that mediate tissue organization and integrity. Subcellular organization, important for asymmetric positioning of different functional domains, also relies on the actin cytoskeleton. Cell shape and elaboration of specialized features such as microvilli and neuronal processes require actin as well. The actin structures involved are often stable features and the filaments within the structures turn over slowly. While it is clear that many of the same proteins important in actin reorganization in motile cells are also involved in differentiation, how actin structures form with the proper organization for their functions and are maintained over long periods of time remains poorly understood.

**Figure 1:**
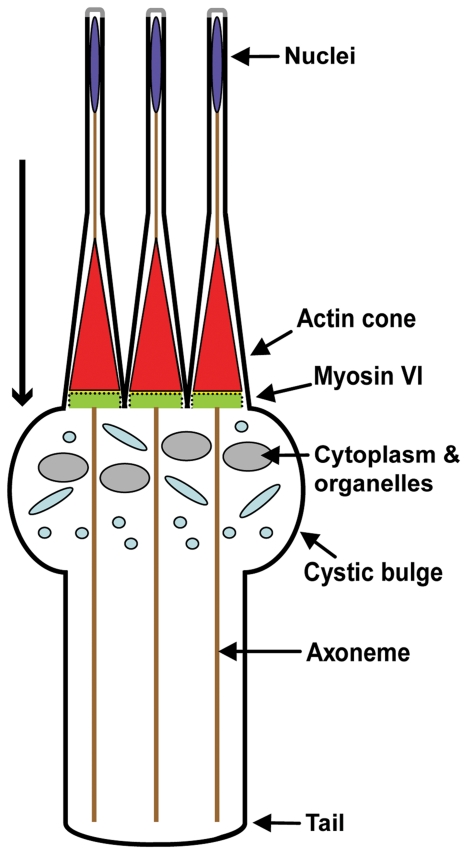
Schematic diagram of spermatid individualization during *Drosophila* spermatogenesis in wild-type animals. Highly elongated cysts of 64 syncytial spermatids (only three are depicted for simplicity) undergo remodeling, to separate the sperm into individual cells. Actin structures called cones (red) travel the length of the elongated cysts, remodeling the membrane and removing cytoplasmic contents as they travel. The removed cytoplasm and organelles accumulate to form the cystic bulge. Actin cones are conical in shape, have myosin VI concentrated at their fronts, and travel synchronously along the axonemes (brown) from the nuclei (blue) to the end of the tail (long arrow).

**Figure 2:**
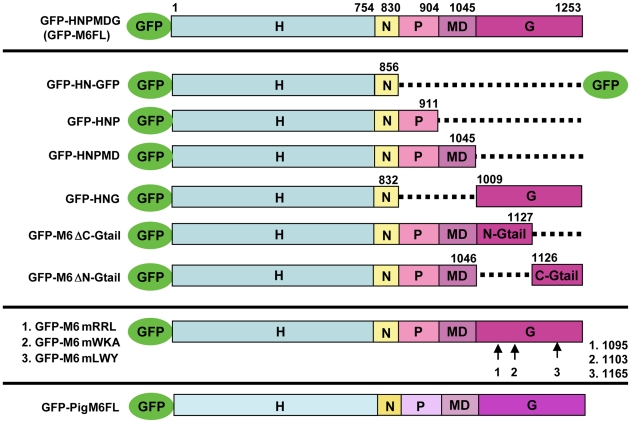
Schematic diagram of engineered versions of myosin VI used in this study. Myosin VI consists of three distinct regions; an NH_2_-terminal motor or head domain (H), responsible for ATP hydrolysis and actin binding; a neck region (N) containing one IQ motif and Insert2 that both bind light chains (calmodulin or androcam, Frank et al., 2006); and tail with three parts: proximal region (P) which forms a three helix bundle [45], Medial and Distal tail (MD) which contains highly charged sequences followed by a single α-helix and globular region (Gtail). The Gtail is well conserved among class VI myosins, and several sequences have been mapped in mammalian myosin VI that are necessary for protein-protein interactions. The domain-deleted molecules used in this study are named according to the regions that remain. For example, GFP-HNG is composed of the head neck and Gtail; the PMD tail regions are deleted. GFP-M6FL is a full length, unmutated, GFP-tagged version of myosin VI that fully rescues fertility in myosin VI mutant animals. Mutant versions in which specific sites were altered (GFP-M6mRRL, GFP-M6mWKA, GFP-M6mLWY) are GFP-tagged full length versions of myosin VI with the specific amino acids as follows: RRL (#1) was mutated to AAA; WKA (#2), WKAKNRKR was changed to WAAANNNR; and LWY (#3), was changed to LLY. Drawings are not to scale.

The process of spermatid individualization in *Drosophila* provides an attractive example for analysis of actin structure formation, maintenance, and function in a specialized cell type. *Drosophila* sperm initially develop as syncytia, but as spermatids mature, each syncytial cell is divided into 64 individual sperm during a process termed individualization. Long-lived actin structures called actin cones mediate the separation of the syncytial spermatids by traveling along the axonemes from the nucleus end to the tip of the tail, removing the cytoplasm and organelles and remodeling the membrane (Figure 1, [1], [2]). The actin cone is made up of two regions: a dense meshwork at the front that excludes cytoplasm and organelles from the sperm cells and parallel bundles at the rear that are important for cone movement. The actin filaments in both regions are oriented such that the minus (slow growing, ‘pointed’) ends face forward relative to the direction of cone movement (i.e., the front of the cones) [3].

Myosin VI is one protein that plays an important role in maintaining actin cone organization as the cones move. Myosin VI localizes to the fronts of the actin cones, where it promotes the formation of the very dense meshwork region [3]. In myosin VI mutants, cones form but do not accumulate sufficient actin. The cones move partway down the axonemes, but fail to exclude cytoplasmic contents [3], [4]. Individualization is disrupted and males are sterile. To understand how this structure is formed, is maintained, and functions during individualization, we are studying myosin VI's mechanism of action.

Myosins are actin-dependent molecular motors that use the energy of ATP hydrolysis to move along actin filaments. Myosin VI moves toward the minus end of actin filaments [5], [6], while all other myosins so far studied walk toward the plus end. Alteration in ATPase kinetics as compared to other myosins [6], [7] leads to some unusual motility behaviors. When backwards force is applied to myosin VI molecules moving along an actin filament in vitro, they stall and remain tightly bound to actin for minutes [8]. This atypical property suggests that myosin VI may serve both as a transporter (similar to other motors) and an anchor.

**Figure 3:**
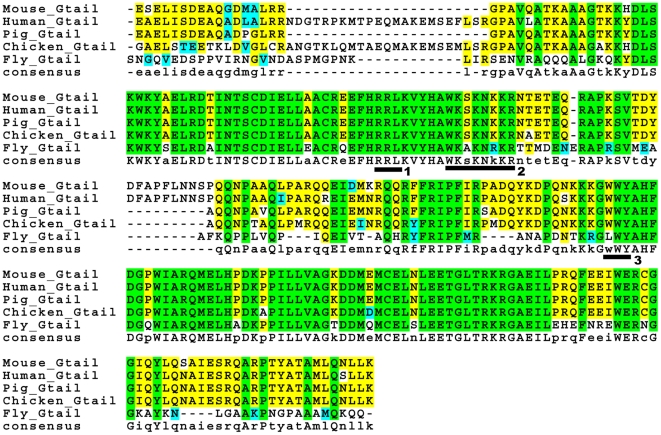
Sequence alignment of the Gtail regions from five species. The RRL (#1), WKA (#2) and LWY (#3) sites are underlined. Green highlighting indicates residues conserved among *Drosophila* and all the vertebrates, blue highlighting indicates conservative substitutions, and yellow highlighting indicates residues conserved among all vertebrates but not with *Drosophila*.

Myosin VI has three major domains (Figure 2): Head, which contains the ATP- and actin-binding sites and is the motor; Neck, which has two light chain binding sites and is important for reverse direction movement; and Tail, which plays a role in stepping and also binds cargoes or adaptors. The tail region has been divided into three main parts [6], [7]: a proximal region (P) that forms a 3-helix bundle, a Medial-Distal region (MD) that is thought to form a single α-helix, and a predicted globular region (Gtail) that contains a number of partner binding sites. Several binding partners that interact with myosin VI's Gtail have been identified in vertebrates (Figure 3), providing important insights into the diversity of myosin VI functions [9], [10], [11], [12], [13], [14].

Myosin VI has been implicated in a large number of different cellular processes, including endocytosis [15], [16], Golgi morphology and secretion [17], [18], basolateral sorting [19], [20], cytokinesis [21], cell movement [22], and adhesion [12], [13]. In these processes, myosin VI may work as a transporter, moving components along actin to the correct sites. Alternatively, it could serve a structural or anchoring role, using its ability to bind tightly to actin under load, in some or all of these processes. [8], [23]

In its role as an anchor, myosin VI would bind stably to actin for long periods, perhaps tethering other cellular components to actin structures. This tethering ability might contribute to actin stabilization in long-lived structures or help keep certain components in particular places on those structures. We proposed that myosin VI's role in actin cone meshwork formation during *Drosophila* sperm development involves an anchor role [3]. Myosin VI may employ anchoring activity in other processes such as epithelial junction maintenance and cell migration. In these cases, myosin VI binds to and is thought to stabilize cadherin/catenin complexes or other adhesion molecules [12], [22], [24]. How myosin VI mediates stabilization and is regulated in these processes remains an open question.

Here, we investigate the mechanisms important for generating a properly structured actin cone. We previously suggested several mechanisms by which a myosin VI anchor might stabilize the cones [3]. First, myosin VI dimers might crosslink filaments in the meshwork, preventing debranching and filament loss. Second, myosin VI binding near the pointed ends of actin filaments might inhibit subunit loss, leading to more stable filaments. Third, myosin VI might bind to and localize proteins important for regulating meshwork formation at the cone front. In our previous work, we provided evidence that myosin VI does not work as a dimer in this process [25], casting doubt on the simplest form of the crosslinking model. In this work, we test the two remaining models by making deleted and mutated versions of myosin VI that alter its function. We demonstrate that both the head and the cargo binding Gtail are important for myosin VI function during spermatid individualization. Furthermore, three conserved sites in the Gtail, previously demonstrated to be necessary for interaction with myosin VI binding partners in mammalian cells, are all required for normal function in actin cone organization. However, the *Drosophila* versions of previously identified mammalian myosin VI binding partners are unlikely to mediate its effects here. In this interesting structure, myosin VI coordinates the activities and localization of actin binding proteins important for assembly and organization of the correct structure.

## Results

### Both Head and Globular tail domains are required for proper myosin VI localization

**Figure 4:**
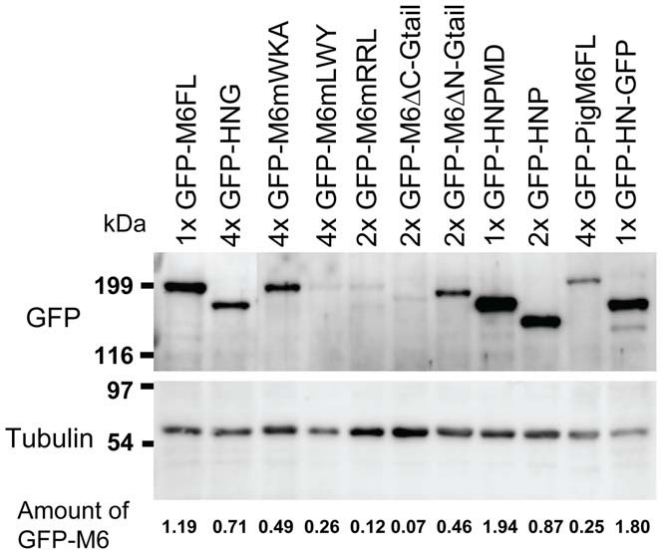
Western blot of testis extracts from flies expressing the indicated molecules. The upper blot was probed with polyclonal anti-GFP antibody and the lower blot with monoclonal anti-α-tubulin antibody. 1×, 2× and 4× indicate the number of copies of the indicated transgene. The GFP band signal intensity was quantitated, standardized to tubulin, and is indicated at the bottom of the blot relative to the amount of endogenous myosin VI (see Materials and Methods). Sizes are indicated in kilodaltons (kDa).

To begin to analyze how myosin VI promotes proper cone organization, we first investigated requirements for localization in a tight band at the cone front. To map the sequences required for proper localization, we introduced a series of GFP-tagged versions of myosin VI, in which particular sequences were deleted or altered (Figure 2), into the genome by P-element mediated transformation. We found that the expression level of different altered myosin VI molecules varied (Figure 4). Expression level in independent lines that expressed the same transgene integrated at different genomic sites varied slightly from each other (not shown), but each altered molecule was expressed at a characteristic level, indicating that this variation is not due to position effects on gene expression in individual lines. Expression levels of some transgenes were significantly less than endogenous myosin VI, suggesting that some engineered molecules were less stable than endogenous myosin VI. We attempted to build lines with multiple transgene copies in which the altered myosin VI molecules were expressed at a level similar to endogenous myosin VI, but in some cases, we could not achieve similar expression (see below).

**Figure 5:**
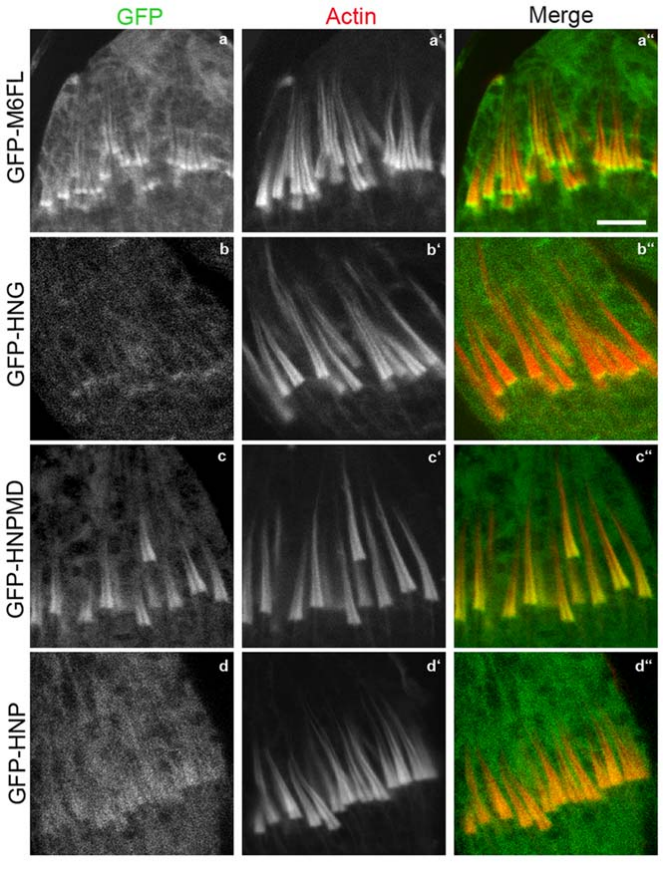
The localization of domain-deleted GFP-tagged versions of myosin VI in wild-type animals. Confocal images of GFP-labeled myosin VI (green), actin (phalloidin; red) and merged images are shown. Bar, 5 µm.

Examination of the various altered versions of myosin VI expressed in wild-type animals (in the presence of endogenous myosin VI) revealed that they fell into three categories: (1) those that localized at the front, (2) those that accumulated all over the cones, and (3) those that did not bind to the cones at all. Full-length wild-type GFP-myosin VI (GFP-M6FL), when expressed in wild-type animals localized at the fronts of actin cones properly (Figure 5a–a″), consistent with our previous findings [26]. A version of myosin VI composed of head (H), neck (N) and globular tail (G) regions, but lacking the proximal (P) and Medial-Distal (MD) tail regions, localized properly (Figure 5b–b″). When only the Gtail (GFP-HNPMD; Figure 5c–c″), or both MD and Gtail (GFP-HNP; Figure 5d–d″), were deleted, localization was abnormal. These deleted versions were enriched on cones but did not accumulate at the front. Instead they were present along the entire length of the cone. Shorter versions, with only the motor/head domain either with or without the neck region (GFP-HN-GFP), did not accumulate on the cones (not shown), despite the fact that they were robustly expressed (Figure 4; Table 1). Additionally, previous work demonstrated that the Gtail alone is unable to localize on cones [3]. Thus, both the head and Gtail are required and together are sufficient for proper front localization. In mammalian cells, although the Gtail domain is sufficient for targeting to some compartments (uncoated vesicles and Golgi), localization to other regions (membrane ruffles, clathrin-coated vesicles or membrane vesicles that participate in cytokinesis) required both the head and Gtail [21], [27], [28], similar to our findings.

**Table 1:** Expression level of and fertility rescue by different versions of myosin VI.

Line	Genotype	protein amount*1	mean number of progeny*2	fertility %*3	n*4
CsprHS83>2× GFP-M6FL	w; CsprHS83 GFP-M6FL-3′UTR; jar1/Dfs87.5e	1.71	ND*7	ND	ND
CsprHS83>1× GFP-M6FL	w; CsprHS83 GFP-M6FL-3′UTR/+; jar1/Dfs87.5e*5	1.19	115.5±4.6	100^a^	36
tubGAL4>>1× GFP-M6FL	w; pUASpGFP-M6FL-3′UTR/+; P{tubP-GAL4}, jar1/Dfs87.5e	0.98	74.86±6.49	64.8^b^	35
tubGAL4>>1× GFP-M6FL	w; pUASpGFP-M6FL-3′UTR/+; P{tubP-GAL4}, jar322/Dfs87.5e	0.84	61.13±7.26	52.9^b^	32
Act5CGAL4>>1× GFP-M6FL	w; pUASpGFP-M6FL-3′UTR/P{Act5C-GAL4}25FO1; jar322/Dfs87.5e	0.28	9.04±3.14	7.8^c^	23
Bab^1^GAL4>>1× GFP-M6FL	w; pUASpGFP-M6FL-3′UTR/+;P{GawB}bab^1^[Pgal4-2], jar1/Dfs87.5e	0.14	14.18±2.75	12.3^c^	45
M6 mutant	w; +; jar1/Dfs87.5e*6	N.A.	0.05±0.04	0.04^d^	37
CsprHS83>2× GFP-HNP	w; CsprHS83 GFP-HNP; jar1/Dfs87.5e	0.87	0.10±0.05	0.1	40
CsprHS83>4× GFP-mWKA	w; CsprHS83 GFP-M6mWKA; jar1/Dfs87.5e	0.49	0.10±0.07	0.1	40
CsprHS83>1× GFP-HNPMD	w; CsprHS83 GFP-HNPMD/+; jar1/Dfs87.5e	1.94	0	0	40
CsprHS83>1× GFP-HN	w; CsprHS83 GFP-HN-GFP/+; jar1/Dfs87.5e	1.80	0	0	40
CsprHS83>4× GFP-ΔN-Gtail	w; CsprHS83 GFP-M6ΔN-Gtail; jar1/Dfs87.5e	0.46	0	0	40
CsprHS83>4× GFP-mLWY	w; CsprHS83 GFP-M6mLWY; jar1/Dfs87.5e	0.26	0	0	40
CsprHS83>2× GFP-mRRL	w; CsprHS83 GFP-M6mRRL/+; jar1/Dfs87.5e	0.12	0	0	40
CsprHS83>4× GFP-ΔC-Gtail	w; CsprHS83 GFP-M6ΔC-Gtail; jar1/Dfs87.5e	0.07	0	0	40
CsprHS83>4× GFP-PigM6FL	w; CsprHS83 GFP-PigM6FL; jar1/Dfs87.5e	0.25	-	2.0	-
CsprHS83>4× GFP-HNG	w; CsprHS83 GFP-HNG; jar1/Dfs87.5e	0.71	-	0.7*8	-

*1 Endogenous myosin VI = 1.

*2 ± means standard error.

*3 Different superscripts indicate that the values differ significantly (p<0.01).

*4 GFP-PigM6FL and GFP-HNG fertility tests were performed in bottles, while the rest were performed by scoring number of progeny in the indicated number of vials.

*5 positive control.

*6 negative control.

*7 ND, not determined.

*8 This fertility test was performed in a previous paper (Noguchi et al., 2009).

### Mapping sequences required for proper front localization of myosin VI

**Figure 6:**
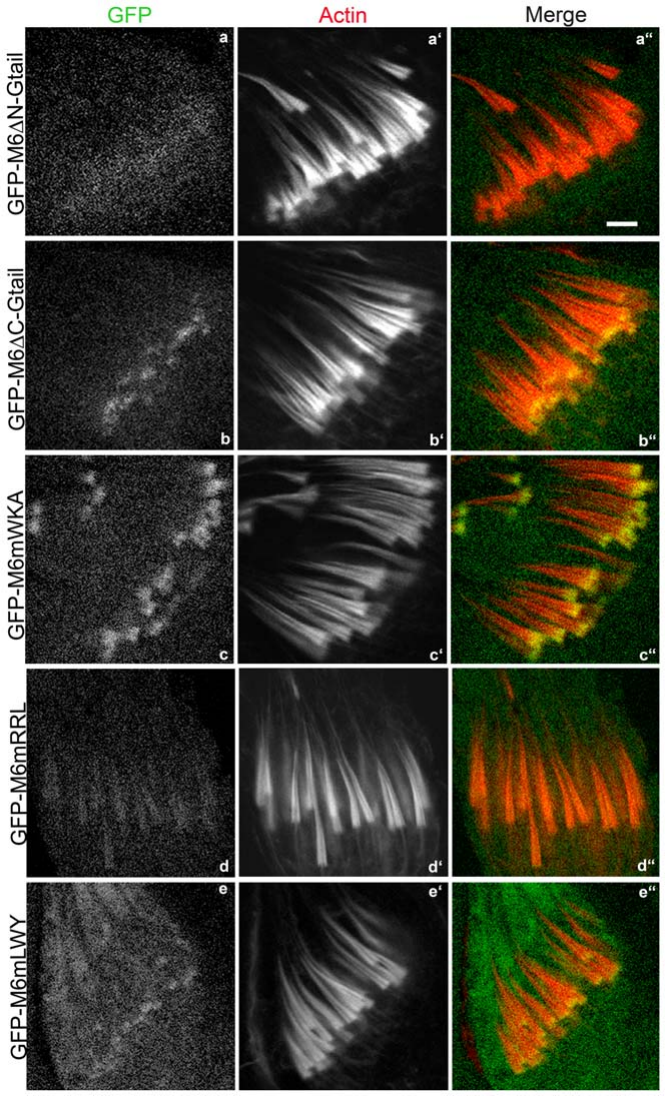
The localization of GFP-tagged myosin VI with mutated or truncated Gtail in wild-type animals. GFP-labeled myosin VI molecules (green), actin staining (phalloidin; red) and merged confocal images are shown. Bar, 5 µm.

Since the *Drosophila* myosin VI Gtail is essential for proper localization to the cone front and previous work on vertebrate myosin VI has identified several interaction partner binding sites which are well conserved in *Drosophila* myosin VI, we asked whether these site(s), were important for localization to the cone fronts. First, we made deletions in which either the N-terminal or C-terminal half of the Gtail was absent (Figure 2). When the N-terminal half of the Gtail was missing (GFP-M6ΔN-Gtail), myosin VI accumulated very slightly on the cones, but was not specifically located at the front (Figure 6a–a″). This distribution was similar to that of myosin VI versions lacking the entire globular tail (GFP-HNP or GFP-HNPMD) described above, although the amount bound is much less than would be expected based on its expression level (46% of endogenous M6; Table 1). This may indicate that this deletion impairs folding of the Gtail. The converse construct, in which the Gtail C-terminal region was deleted (GFP-M6ΔC-Gtail), was properly localized (Figure 6b–b″), although the fluorescence signal observed is weak, due to the low expression level of this molecule (7% of endogenous M6).

The above results suggested that the N-terminal region of the Gtail was important for front localization. Therefore, we mutated two partner-binding sites in this region that are conserved between mammalian and *Drosophila* myosin VI. Expression of myosin VI with mutations in the sequences corresponding to the PtdIns(4,5)P2 (PIP2)-binding WKA site (GFP-M6mWKA), which is important for clathrin-coated vesicle interaction [10], resulted in normal localization (Figure 6c–c″). In contrast, when the sequence corresponding to the GIPC-binding RRL site (GFP-M6mRRL), involved in endocytic uncoated vesicle trafficking [29], [30], was mutated, myosin VI was abnormally distributed all over the cones (Figure 6d–d″).

The Gtail C-terminal region has a conserved site, LWY, at which Dab2, a protein involved in endocytosis, binds [31], [32]. When this site was mutated (GFP-M6mLWY), myosin VI was properly localized (Figure 6e–e″). This is consistent with the finding that removal of the C-terminal region of the Gtail had no effect on myosin VI localization. Together these results demonstrate that the N-terminal region of the Gtail is necessary for proper localization and the RRL site is critical in this region.

### Rescue of defects using myosin VI transgenes is dose-dependent

**Figure 7:**
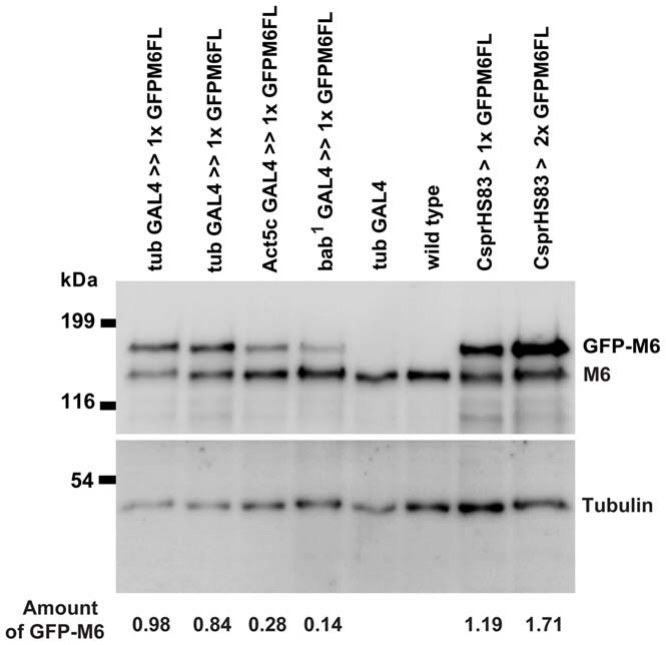
Western blot of testis extracts from flies expressing the indicated molecules. The upper blot was probed with monoclonal anti-myosin VI antibody (3c7) and the lower blot with monoclonal anti-tubulin antibody. The upper GFP myosin VI band signal intensity was quantitated, standardized to the endogenous myosin VI band in each lane, and is indicated at the bottom of the blot, relative to the amount in GFP-M6FL. Sizes are indicated in kilodaltons (kDa).

In previous work [3], we showed that the amount of actin in cones is proportional to myosin VI amount. However, some altered versions of myosin VI were able to fully rescue actin cone shape and size when expressed at levels much lower than endogenous myosin VI [25]. Since many of the myosin VI constructs used here were expressed at low levels, we determined the dose-dependency of rescue by wild-type myosin VI to control for this variation. At the lowest level of expression, GFP-myosin VI driven using bab^1^ GAL4 (14% of endogenous myosin VI amount) or act5C GAL4 (28%) (Figure 7, Table 1) resulted in complete rescue of actin cones (Figure 7). Rescue of fertility was significant but incomplete (8–12% progeny) compared to the control, GFP-M6FL driven by CsprHS83 (expressed at 120% of endogenous). All of the altered transgenes we analyzed for rescue of myosin VI mutant effects on the cone size and shape were expressed at levels equal to or greater than 14% (see Figure 7, Table 1), so if the mutated versions functioned as well as wild type, we should observe complete cone rescue and partial fertility recovery. If rescue was less robust, we can conclude that the mutated version did not function properly.

### Proper localization of myosin VI is required for correct actin cone shape and size

**Figure 8:**
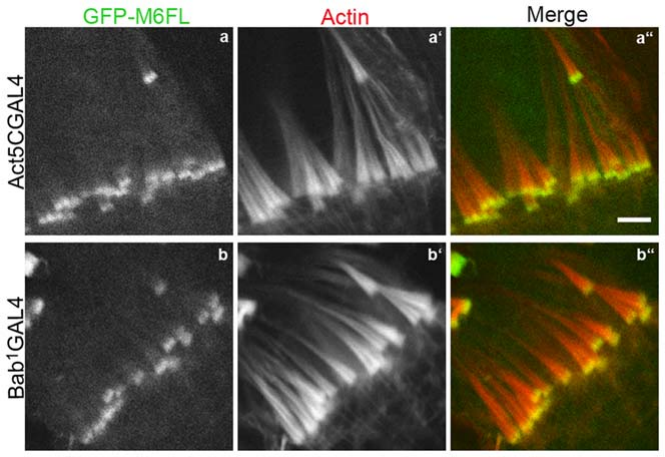
Effects on actin cone shape and size when myosin VI is expressed at low levels. Confocal images of GFP-labeled myosin VI (green), actin (phalloidin; red) and merged images are shown. Driving expression with ActGAL4 or babGAL4 results in expression of GFP-myosin VI at 28% or 14%, respectively, of the level of endogenous myosin VI. Bar, 5 µm.

**Figure 9:**
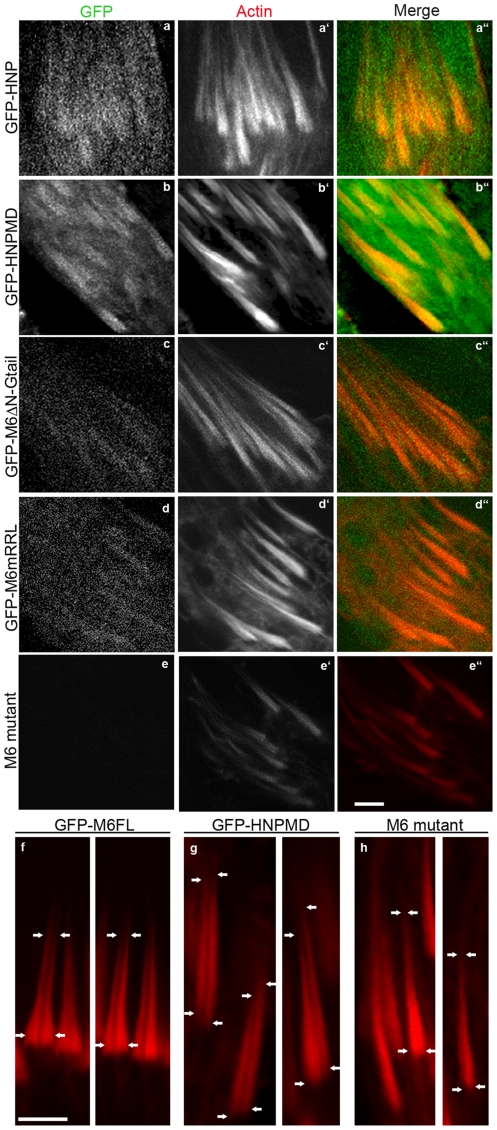
Effect of altered versions of myosin VI on actin cone formation in myosin VI mutant animals. Confocal images of GFP-myosin VI (green), actin (red), and merged images are shown. Examples of altered versions that were enriched on cones but uniform along their length are shown. In panels f (GFP-M6FL), g (GFP-HNPMD), and h, (M6 mutant) higher magnification images of actin cones by phalloidin staining are shown to better illustrate altered shapes. Arrows indicate position of front and back of the actin cones in symmetrical (f and h) and asymmetric (g) cones.

**Figure 10:**
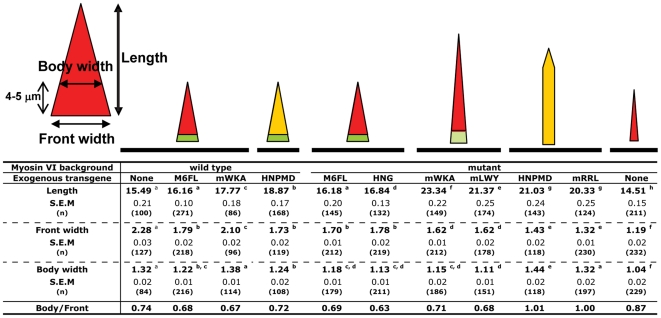
Expression of mutated or truncated myosin VI altered actin cone shape. The diagram on the left shows the measurements performed to compare cone shapes. All values are in µm. At the top of each row, the shape (actin – red) and myosin VI distribution (green) is depicted in schematic form. Yellow indicates where actin and myosin VI overlap. S. E. M, standard error of the mean. For each attribute measured (length, front width, and body width) data from all lines were compared in pairwise combinations. Those values that did not differ significantly are indicated with the same superscript letter. Those values that differed significantly (p<0.01) are indicated with different superscript letters.

To determine if front localization was necessary for myosin VI to properly function in actin assembly and organization, we examined whether the mutant/deleted forms of myosin VI that were localized uniformly along the entire actin cone were able to rescue myosin VI loss-of function effects on the cones. When the molecules that lacked the Gtail (GFP-HNPMD) or the N-terminal half of the Gtail (GFP-M6ΔN-Gtail), or in which the conserved Gtail site important for front localization (GFP-M6mRRL) was mutated were expressed in myosin VI mutant testes (Figure 8), the resulting actin cone shapes were very different than seen when molecules that localized to the front, such as GFP-M6FL or GFP-HNG [25] were expressed. By measuring length, width at the front, and width approximately 1/3 of the way along the cone length (body width), we could quantitatively assess cone size and shape. Cones from flies expressing GFP-HNPMD or GFP-M6mRRL were almost cylindrical (rather than conical; front/body width ratio of 1.0 vs. 0.87 for GFP-HNG; Figure 9) and sometimes appeared slightly wider in the middle than at the front (Figure 9g). Additionally, the front border was not flat, but often rounded or ill-defined (Figure 9a–d″, g). The cones were larger (Figure 10) and stained more brightly with phalloidin than cones in the myosin VI mutant (Figure 9). Their fronts were intermediate in width between myosin VI mutant and wild-type cones and they were longer than cones in wild type (20–21 µm vs. 16–17 µm; Figure 10). Interestingly, when the version lacking the Gtail (GFP-HNPMD) was expressed in wild-type animals, cone length and body width were increased compared to cones in wild-type animals that expressed GFP-M6FL (Figure 10). This mislocalized myosin VI was able to change actin cone shape even when endogenous myosin VI was present. Therefore, accumulation of myosin VI all over the cone caused abnormal actin accumulation and organization. From these observations, we conclude that proper localization of myosin VI at the cone front is important for generating normal actin cone shape and filament organization.

**Figure 11:**
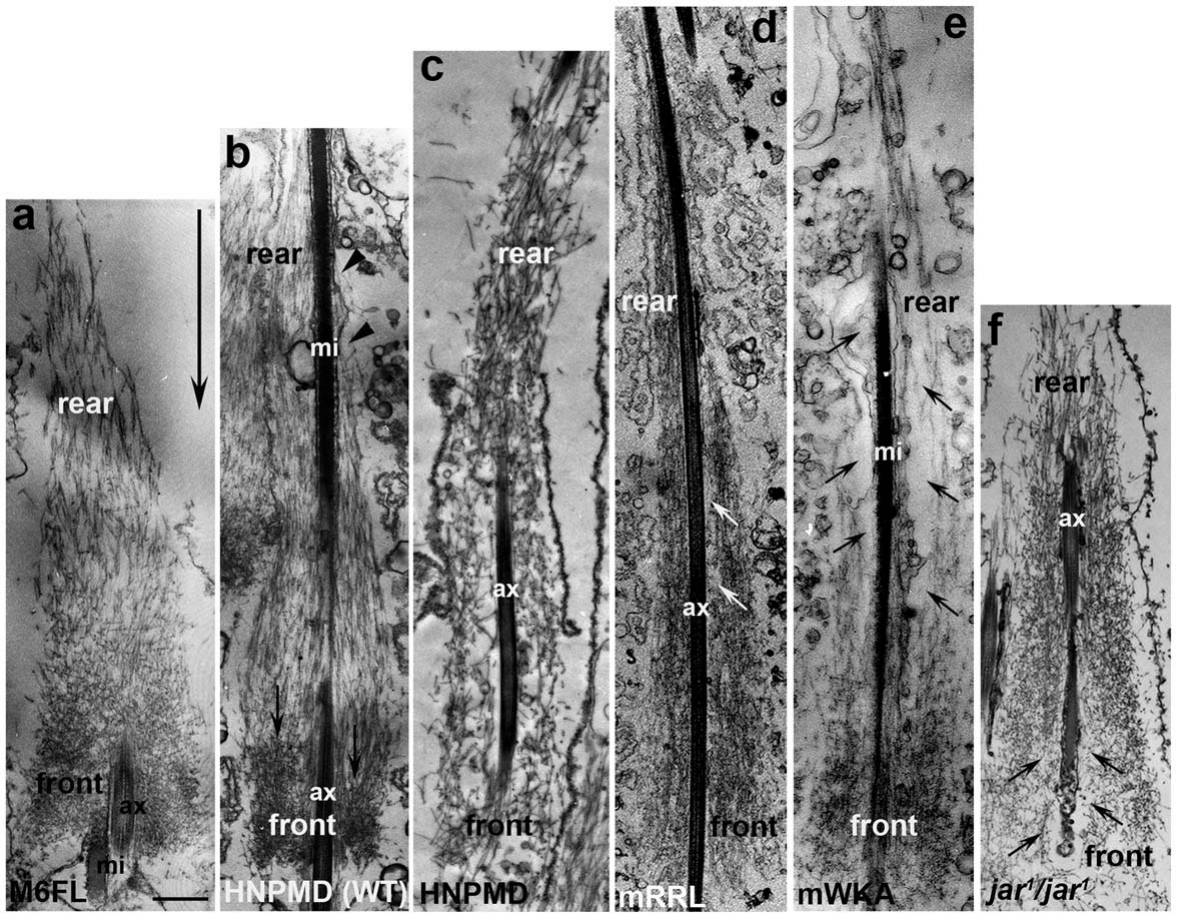
EM images of actin cones at late stage. The indicated transgene was expressed in wild type (b) or myosin VI mutant (a, c–f) background. (a) GFP-M6FL (b) GFP-HNPMD (Gtail deleted) expressed in wild-type animal; (c) GFP-HNPMD expressed in a myosin VI mutant animal; (d) GFP-M6 mRRL in a myosin VI mutant animal, (e) GFP-M6mWKA in a myosin VI mutant animal; (f) no transgene (*jar* ^1^ mutant) Large arrow in (a) indicates the direction of cone movement, small arrows indicate areas near the cone center that lack actin filaments. mi, mitochondria; ax, axoneme. Bar, 1 µm.

Close examination of actin cones at high magnification revealed an effect of GFP-HNPMD that suggested mislocalized myosin VI disrupted actin cone structure. These cones appeared asymmetric, with misaligned fronts and rear borders (note arrows at front and back of cone on each side; Figure 9g). Sometimes more actin staining was observed on one side of the axoneme (Figure 9g, cone on the right panel; see also Figure 11b and Figure S2). By comparison, when GFP-M6FL was expressed, the actin structures were conical, with wide fronts, and the axoneme ran down the middle (Figure 9f). When no myosin VI was present, cones were thin and had a rounded front (‘tear-drop shaped’), but were symmetrical (Figure 9h).

### Mislocalized myosin VI disrupts cone structure

To better understand the disruptive effects of mislocalized myosin VI, we examined actin filament organization using myosin II S1 decoration and electron microscopy. Previously, we showed that actin cones are composed of two regions, parallel bundles at the rear and a dense meshwork at the front, as seen in cones from wild type [3], [33] and GFP-M6FL expressing animals (Figure 11a). The meshwork at the front gives the cones their conical shape. In the myosin VI mutant, cones had significantly fewer parallel bundles and sparse meshwork. Often space without actin filaments was visible around the axoneme (Figure 11f, black arrows). When a deleted myosin VI molecule that is distributed along the whole length (GFP-HNPMD, lacking the Gtail; Figure 11c) was expressed in myosin VI mutant animals, the cones looked similar to myosin VI mutant cones (Figure 11f ), with less filaments than are present in wild type and looser overall structure. However, unlike the myosin VI mutant cones, the domains of parallel bundles and meshwork were not apparent; instead the filaments throughout the cones were oriented at all angles and the actin appeared less dense (Figure 11c; more examples and high-magnification images are shown in Figure S1). When myosin VI was mislocalized all along the cone in wild-type animals [both endogenous myosin VI and GFP-HNPMD (Figure 11b], the actin filament organization was altered significantly. Dense meshwork was visible at the front of the cones (black arrows), presumably due to the properly localized endogenous myosin VI, but the middle of the cones appeared swollen and less dense. Moreover, the actin cones were often asymmetric (Figure 11b; more examples are shown in Figure S2), with more actin on one side of the axoneme. We have never observed this asymmetric cone phenotype in wild type or myosin VI mutant cones.

To determine if asymmetric actin accumulation was a common effect of having myosin VI mislocalized all over the actin cones, we examined actin cones from animals that expressed myosin VI with the Gtail site required for front localization mutated (GFP-M6mRRL; Figure 11d). We observed slightly asymmetric actin cones and the distinct regions of meshwork and bundles seen in both normal and myosin VI mutant cones were not apparent. These effects were weaker than the effects of expression of the Gtail deleted version, perhaps due to the very low expression level of GFP-M6mRRL (Figure 4; 12% of endogenous M6). Together these results strongly argue that front localization of myosin VI is critical for proper function.

### Is localization of myosin VI to the cone front sufficient for proper function?

Previously, we proposed two ideas for how myosin VI might regulate cone actin content and organization using its anchoring ability and localization at the cone fronts where the pointed ends of the actin filaments are located. One possibility is that binding of myosin VI might be sufficient to slow pointed end depolymerization. Alternatively, myosin VI's role might be to localize binding partners that mediate effects on actin assembly and organization. If the first model is correct, we would predict that front localization would be sufficient for rescue activity. We tested this idea by analyzing actin cone shape and size in myosin VI mutant animals expressing the altered myosin VI molecules that were capable of localizing to the cone fronts. We found that localization at the cone front was not sufficient for cone rescue (see below). Some mutant myosin VI molecules in the front localizing group rescued well, while others did not.

**Figure 12:**
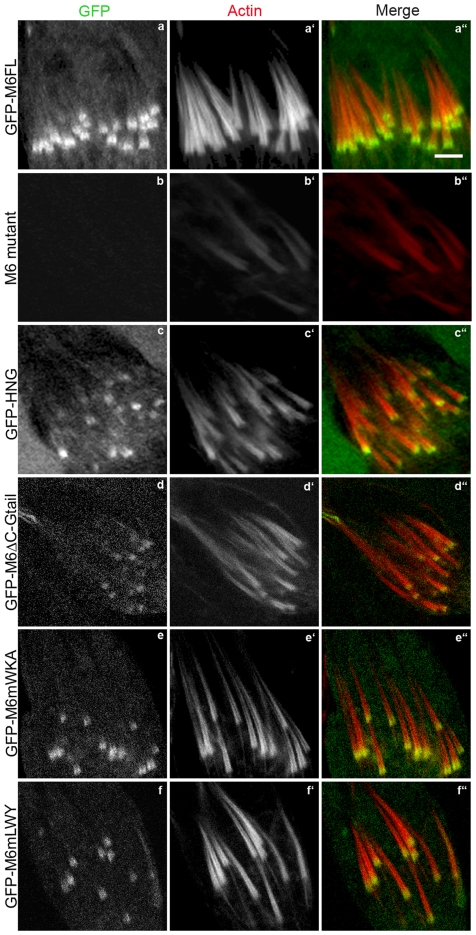
Changes in actin cone shape and size caused by mutant forms of myosin VI that localize to the cone fronts. Confocal images of GFP-labeled myosin VI (green), actin (phalloidin; red) and merged images are shown. Bar, 5 µm.

The molecules that localized and rescued well included the Head, Neck and Gtail [full-length GFP-myosin VI (GFP-M6FL; Figure 12a–a″) and the deleted version GFP-HNG, (Figure 12c–c″.)] In these cases, the actin cone structure was significantly rescued with robust actin staining, and the characteristic conical shape. For GFP-HNG and full-length GFP-myosin VI, cone length (16–17 µm), width at the front (1.7–1.8 µm) and the ratio of front to body width (0.6–0.7) were nearly identical (Figure 10). Moreover, the cones were better aligned during movement than in the myosin VI mutant (Figure 12b–b″) and fertility was also partially or completely rescued (Table 1). The low level of fertility rescue by GFP-HNG is due to a requirement for myosin VI at a stage after cone movement and cytoplasmic exclusion during individualization. GFP-HNG fails to rescue this late defect [25]. These data confirm that versions of myosin VI with only Head, Neck and Globular tail (GFP-HNG) can replace myosin VI in actin cone formation and function.

Three mutated molecules, GFP-M6ΔC-Gtail, GFP-M6mWKA and GFP-M6mLWY, localized at the front but did not function normally (Figure 12d–f″). In these molecules Gtail sites corresponding to those required for mammalian myosin VI interaction with several binding partners were mutated or removed. Cone shape was analyzed in greater detail for two of these molecules, GFP-M6mWKA and GFP-M6mLWY. When expressed in a myosin VI mutant animal, shape was conical (Front to body ratio of ∼0.7; Figure 10) and synchrony of cone movement was better than in the myosin VI mutant, but the cones were abnormal (Figure 12d–f″; Figure 10). Cones were much longer (23.34 and 21.37 µm respectively) than cones in either wild type (15.49 µm) or animals that expressed GFP-M6FL (16.6 µm). Cones were wider than in the M6 mutant (1.62 vs. 1.19 µm), but not as wide as cones in animals that expressed GFP-M6FL (1.79 µm). Despite being localized and affecting cone structure, expression of these transgenes did not rescue fertility at all (Table 1). Expression of GFP-M6FL at comparable levels was sufficient for good cone shape rescue (Figure 8) and partial rescue of fertility (Table 1). We conclude that these two sites must be important for myosin VI's proper function, perhaps via interaction with binding proteins that mediate myosin VI's effects on meshwork formation or organization. Front localization of a functional motor alone is not sufficient for normal function.

**Figure 13:**
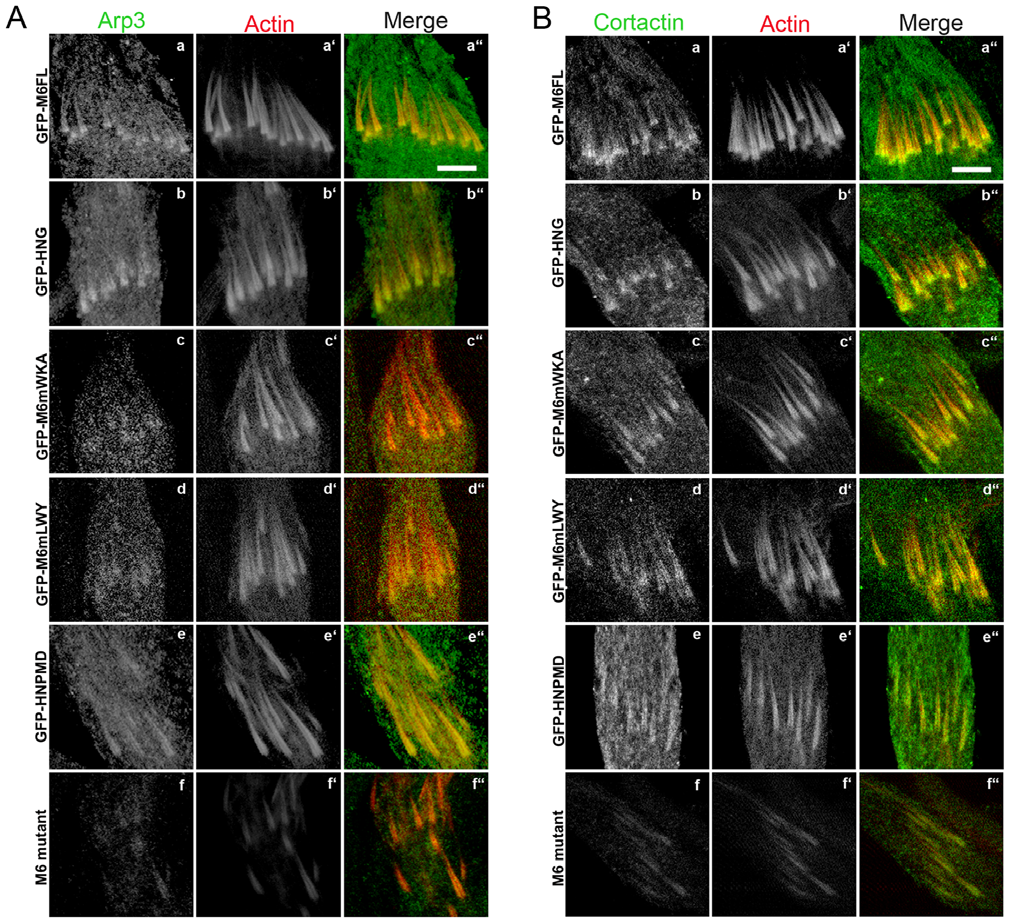
Arp3 and cortactin accumulation were influenced by myosin VI localization. False-colored confocal images of Arp3 (A, green) or cortactin (B, green), actin (phalloidin, red) and merged images in a myosin VI mutant background are shown. GFP-myosin VI is not shown in this figure Bar, 10 µm.

**Figure 14:**
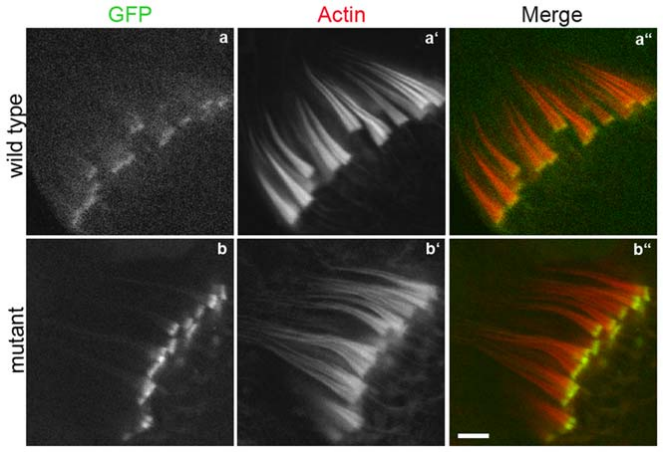
Localization of and rescue by the mammalian myosin VI orthologue, GFP-pigM6FL. The transgene was expressed in wild type (a–a″) and myosin VI deficient animals (b–b″). Bar, 5 µm.

We hypothesized that the failure of GFP-M6mWKA and GFP-M6mLWY to rescue actin cone shape despite their proper localization was due to the inability of these molecules to affect the distribution of actin assembly regulators. We tested this idea by examining the distribution of the Arp 2/3 complex (Figure 13) and cortactin (Figure 14). The Arp 2/3 complex and cortactin were enriched in the meshwork regions in distributions broader than myosin VI [3], [26], Figure 13a–a″, Figure 14a–a″). These enrichments were lost when myosin VI was absent ([26] and Figure 13f–f″, Figure 14f–f″). When functional myosin VI was present (GFP-FL-Myosin VI, Figure 13a–a″; Fig. 14a–a″; GFP-HNG, Figure 13b–b″; Figure 14b–b″), Arp3 and cortactin were clearly visible and enriched in the front region of the cone that corresponds to the meshwork region. However, when front-localized but abnormally functioning myosin VI molecules were expressed (GFP-M6mWKA; Figure 13c–c″ and Figure 14c–c″ and GFP-M6mLWY; Figure 13d–d″; Figure 14d–d″), Arp3 was visible on the cones in a broader than normal region at the front and was barely detected above background. Cortactin was clearly present, but distributed along the entire cone length. The lack of robust recruitment and proper localization of these two actin regulators supports the idea that the two conserved sites, WKA and LWY, are important for myosin VI to regulate actin organization. The mislocalization of actin regulators along the length of the cone could explain the increase in length (see Discussion). However, it is unlikely that the Arp 2/3 complex or cortactin directly bind to these sites, because their distributions on wild-type cones are much broader than that of myosin VI.

The organization of filaments at the EM level in myosin II-S1 decorated preparations was observed to determine how these altered versions of myosin VI affected filament organization. Very long and thin actin cones that often lacked a dense front meshwork were observed (GFP-M6mWKA; Figure 11e). No clear domains of meshwork and bundles were usually discernable. Additionally, most of the actin filaments in the rear region failed to form bundles and instead were loosely arranged. Empty space was observed around the axoneme (Figure 11e, small arrows). These results support the idea that front localization of the myosin VI motor is not sufficient and additional interacting molecules mediate myosin VI's effect on actin cones.

### Vertebrate myosin VI can functionally substitute for *Drosophila* myosin VI

Since several conserved sites in the Gtail required for interaction with myosin VI binding partners in mammals are also required for *Drosophila* myosin VI localization and function, we wondered whether porcine myosin VI [34] would be able to substitute for *Drosophila* myosin VI. When expressed during *Drosophila* spermatogenesis, the pig myosin VI (GFP-PigM6FL) localized properly on the front of actin cones and rescued cone actin content and shape well (Figure 14). In addition, fertility was partially rescued (Table 1). The strength of rescue was similar to that seen when *Drosophila* myosin VI was expressed at similar levels (Table 1). We conclude that pig myosin VI can substitute for fly myosin VI in actin cone formation.

## Discussion

In this study we tested several models of myosin VI's mechanism of action in promoting proper actin cone organization and structure. The data presented here shows that localization of myosin VI to the fronts of the cones is necessary but not sufficient to rescue cone shape. This result argues against a model in which binding of myosin VI near the pointed ends inhibits depolymerization, leading to more actin in the meshwork. Our previous work showed that sequences important for dimerization (medial and distal tail) were not required for myosin VI to stabilize the cones arguing against a crosslinking model [25]. Instead, the model most consistent with our data is that myosin VI localizes and tethers molecules that, in turn, regulate actin assembly or organization.

Mapping of sequences important for myosin VI's role in formation of actin cones revealed that localization and effects on actin organization and content are separable activities and two different inputs are needed. First, myosin VI must be localized at the cone front and this localization requires a conserved site, RRL, in the Gtail as well as the head (motor) domain. We hypothesize that an RRL site-binding partner is important. However, a second input, mediated by two other Gtail sites, WKA and LWY, is also important. When mutant versions of myosin VI that have an altered WKA or LWY site were expressed, they were at the cone fronts, but they did not rescue myosin VI function. We hypothesize that molecules that regulate actin assembly and/or cone structure bind to the WKA and LWY sites. Although myosin VI clearly affects the distribution of cortactin and Arp 2/3 complex, their proteins' distributions are not coincident with myosin VI [26], indicating direct binding of these actin regulators at the WKA or LWY sites is unlikely. We hypothesize that the binding sites must interact with some regulator(s) that affects Arp 2/3 complex and cortactin function.

Our hypothesis that interactions with binding partners at these three conserved sites mediate the localization and function of myosin VI on the cones is the most straightforward explanation of our results. However, in the absence of information about the identity of such partners, we cannot rule out the possibility that the Gtail promotes actin cone formation and function through a mechanism other than cargo binding.

Some of the mutant versions of myosin VI used in this study were expressed at levels much lower than endogenous myosin VI and, in some cases, we were unable to increase their expression level significantly by increasing transgene copy number. Since strength of rescue is proportional to myosin VI amount (Table 1; Figure 7, 8), we controlled for this variation in level by quantitating rescue by various amounts of unmutated GFP-tagged myosin VI. All the mutant molecules used in the quantitative analysis of cone shape and size were expressed at levels equal to or higher than the lowest level of GFP-M6FL (14%) that produced complete cone rescue.

The low level of protein accumulation may indicate that these molecules are unstable, possibly due to improper folding. We cannot rule out the possibility that the sequence change at RRL leads to improper folding that is equivalent to Gtail deletion. However, two of the mutated versions, GFP-M6mWKA and GFP-M6mLWY, localize normally on the cone fronts. Thus, they retain enough structure in their G-tails to have intact sites for localization. Improper Gtail folding seems like an unlikely explanation.

The conserved binding site, RRL, previously characterized in mammalian myosin VI, is critical for myosin VI localization on cones. A large number of mammalian myosin VI interaction partners require the RRL site to bind. Of those proteins (GIPC, optineurin, SAP97 and T6BP/NDP52), orthologues of only GIPC and SAP97, called Kermit and Discs large 1 (Dlg), respectively, are present in the *Drosophila* genome. We stained with a Dlg (SAP97 orthologue) antibody, but failed to see any localization on actin cones (not shown). Fly strains that express siRNA for these proteins under the control of a GAL4 promoter were obtained (VDRC, [35]), and the siRNAs were expressed using a GAL4 driver that is expressed in the male germ line at an early stage (Bam-GAL4). We saw no effect on fertility or actin cone morphology (data not shown). As a control, we expressed siRNA to myosin VI in a similar manner. This resulted in male sterility as expected, suggesting that the Bam-Gal4 driver is able to drive expression of siRNA to levels sufficient to severely deplete some proteins during individualization. Recently, *Drosophila* Kermit (GIPC orthologue) mutants were isolated [36]. Male Kermit mutants were fertile, suggesting that Kermit is not the relevant myosin VI binding partner here.

We also investigated Dab, the *Drosophila* orthologue of Dab2, a previously identified binding partner for mammalian myosin VI at the LWY site [31], [32]. We failed to see any localization of Dab on actin cones and animals expressing Dab siRNA in the testis were fertile with no actin cone defects (not shown). However, we could not confirm that Dab proteins levels were reduced. Recently, identification of binding partners for *Drosophila* myosin VI using immunoprecipitation and mass spectrometry [37] failed to identify Dab. Thus, Dab seems unlikely to be the relevant binding partner in this process.

The WKA site in mammalian myosin VI mediates interaction with phospholipids, including PIP2. PIP2 and skittles, a PIP2 biosynthetic enzyme, localize to the growing tip of the elongating cysts. Depletion of PIP2 using ectopic expression of the PIP2 phosphatase, SigD, or loss of skittles function leads to failure of cysts to polarize and elongate [38]. These early defects prevent observation of effects on individualization. We attempted to deplete PIP2 later in development to test for individualization defects by expressing siRNA that targets skittles. No effects on individualization were observed (not shown), but we could not confirm that PIP2 levels were affected by our manipulation.

Mislocalized myosin VI exerts an ectopic effect on cone structure, indicating proper localization is a key feature of proper function. Mistargeting of myosin VI ectopically all over the cone interfered with normal cone formation even when endogenous myosin VI was present. In addition, in normal cones, myosin VI's presence at the front mediates formation of a dense meshwork, but when truncated myosin VI is present all over the cones, there is no ectopic dense meshwork in the rear. Another effect of mistargeted myosin VI is asymmetric actin accumulation. This ectopic effect on actin organization suggests that restricting myosin VI to its domain at the front is an important feature of its *in vivo* function.

Myosin VI is a highly conserved protein (*Drosophila* and mammalian myosin VI are ∼70% similar). Mammalian and *Drosophila* myosin VI have been implicated in some of the same cellular processes, such as basolateral sorting/localization, adhesion, and cell movement (see Introduction). Conserved sites in the Gtail (Figure 2) previously implicated in mammalian cells for myosin VI function are also required for myosin VI function in actin organization in *Drosophila* (this work). Furthermore, pig myosin VI can rescue actin cone structure well (this work), providing strong evidence that the features, interactions, and properties of *Drosophila* myosin VI are similar to those that have been characterized in mammalian myosin VI. It is likely that the regulation of actin assembly and organization in cones relies on myosin VI properties similar to those used by mammalian myosin VI *in vivo*. While actin stabilization or structure modification has not been directly demonstrated for mammalian myosin VI, there are several processes in which actin structure regulation might be important. One such example is cochlear hair cell stereocilia maintenance [39]. Myosin VI is enriched at the base of the stereocilia, in the terminal web region, which is composed of dense actin meshwork. The actin bundles that form the core of the stereocilia are anchored in the terminal web meshwork [40], [41]. When myosin VI is absent, the stereocilia degenerate [39]. In addition, myosin VI binds to several cell adhesion proteins and plays a role in stabilizing cell-cell and cell-substrate junctions [12], [13], [22], [42]. Actin filaments interact with these adhesion sites, raising the possibility that myosin VI regulates actin assembly and/or organization in this case as well. Further studies of these and other processes in *Drosophila* and in mammalian cells are needed to determine if myosin VI works by regulating actin organization via interactions with its Gtail.

The experiments reported here provide significant new insight into myosin VI's mechanism of action *in vivo*. The identification of specific sequences in the Gtail required for myosin VI to function properly and the knowledge that localization and effects on actin are separable activities suggests the specific, testable hypothesis that myosin VI localizes a regulator (or regulators) that affect actin organization. Novel interaction partners are likely to be involved, since we were unable to implicate the previously identified proteins that bind to these sites. An important challenge for the future will be to determine the identity of the binding partners.

## Materials and Methods

### Transgene Construction

GFP-M6FL and GFP-HNG were used previously [25]. The transgene constructs GFP-HNP, GFP-M6ΔC-Gtail, GFP-M6ΔN-Gtail, GFP-M6mRRL, GFP-M6mWKA and GFP-M6mLWY, were made using the strategy described in Noguchi et al., 2009. The deleted or altered regions are as follows: GFP-HNP; 911–1253 (LNT-KQQ), GFP-M6ΔC-Gtail; 1127–1253 (AFK-KQQ), GFP-M6ΔN-Gtail; 1046–1126 (IRS-MEA), GFP-M6mRRL; 1095–7 (RRL→AAA), GFP-M6mWKA; 1103–1110 (WKAKNRKR→WAAANNNR), GFP-M6mLWY; 1165–1167 (LWY→LLY). GFP-HN-GFP and GFP-HNPMD were made by standard PCR and QuikChange (Stratagene, La Jolla, CA) methods. The HN fragment is the sequence from the start ATG to the end of the Neck region (856, IAS). The GFP-HNPMD fragment is the sequence from the initiating Met to amino acid 1045 (MGP)]. GFP-pig M6FL was made by amplifying from plasmid pEGFP-C1 Myo6 full [43]. A G to A mutation that created a Gly to Ser amino acid change was identified in the original vector and was corrected by QuikChange. The PigM6FL sequence was inserted with GFP and the fly myosin VI 3′ UTR in the vector CsprHS83-3′. For all constructs, PCR-amplified fragments were cloned into TA vector (Invitrogen, Carlsbad, CA) and the sequences and junction sites were confirmed. Transformant flies were generated by Genetic Services (Cambridge, MA).

### Fly husbandry and crosses

Flies were raised on standard cornmeal agar medium at 25°C and Oregon R was used as the wild-type strain. To examine transgenes in a myosin VI null background in testis, the following genotypes were generated: w; P[w+transgene]/+; *jar* ^1^/Df(3R)S87.5e (one copy), w; P[w+transgene]/P[w+transgene]; *jar* ^1^/Df(3R)S87.5e (two copies), or w; P[w+transgene] P[w+transgene]/P[w+transgene] P[w+transgene]; *jar* ^1^/Df(3R)S87.5e (four copies). Flies bearing two or four copies of the transgenes were generated by crosses or recombination to increase protein expression.

### Expression level determination

Relative expression levels were determined by Western blot as described previously [25] except that 2× protein sample buffer with 0.2 M DTT was used and the equivalent of two testes per lane were loaded on a 7.5% polyacrylamide gel. The top halves of the blots were probed with rabbit anti-GFP antibody (Clontech) and the bottom halves were probed with mouse monoclonal antibody DM1A to detect α-tubulin (Simga-Aldrich, St. Louis, MO). Detection was performed using Super Signal West Femto (Pierce Chemical, Rockford, IL), and chemiluminescence was captured and quantified using a Fuji Film LAS-1000 imager and ImageGuage software (Fujifilm, Tokyo, Japan). As a standard, the amount of GFP-M6FL and endogenous myosin VI were measured in a strain with the GFP-M6FL transgene in a wild-type background by blotting and probing with monoclonal anti-myosin VI antibody (3c7, see Figure 7). The relative amount of protein expressed from each GFP-tagged transgene was determined by probing with anti-GFP antibody, standardized to tubulin amount. The amount relative to endogenous myosin VI was calculated using the previously determined ratio of the amount GFP-M6FL to endogenous myosin VI (1.19∶1, see Figure 7).

### Fertility assays

In Table 1, fertility of myosin VI mutant lines expressing various transgenes was quantitated. We used HS83 promoter-GFP-Myosin-VI full length (CsprHS83 GFP-M6FL-3′UTR), and UAS -GFP-Myosin-VI full length (pUASpGFP-M6FL-3′UTR) crossed with several GAL4 drivers to obtain different levels of GFP-myosin VI expression. All of the deleted/mutated versions of myosin VI that were analyzed were also tested. To examine transgenes in a myosin VI null background in testis, flies of the following genotypes were generated: w; P[w+transgene]/+; *jar* ^1^/Df(3R)S87.5e (one copy), w; P[w+transgene]/P[w+transgene]; *jar* ^1^/Df(3R)S87.5e (two copies), or w; P[w+transgene] P[w+transgene]/P[w+transgene] P[w+transgene]; *jar* ^1^/Df(3R)S87.5e (four copies). In some cases, the *jar* ^322^ instead of *jar* ^1^ allelle was used. This genotype also is male sterile in combination with Df(3R)S87.5e. To increase protein expression, animals bearing two or four copies of the transgenes were generated by crosses or recombination. Myosin VI mutant (*jar* ^1^/Df(3R)S87.5e) served as a negative control. The number of progeny was compared to that obtained with HS83 promoter-GFP-Myosin-VI full length (w; CsprHS83 GFP-M6FL-3′UTR/+; *jar* ^1^/Df(3R)S87.5e, 100%, Table 1). Ten young males (1–3 days old) of the test genotype were placed with 5 young wild-type virgin females in a vial supplemented with moist Instant *Drosophila* media (Carolina Biological Supply, Burlington, NC) and a piece of Kimwipe (Kimberly-Clark, Roswell, GA) (day 0). After 7 days at 25°C, adults were removed. Progeny in each vial were counted until day 18. The number of vials counted is shown for each genotype and the average number of progeny per vial is reported. For the GFP-HNG fly line, the fertility data were reported in [25]. GFP-PigM6FL line was tested in a similar manner except that 25 virgin wild-type females and 10 males were used and the crosses were done in bottles. We performed statistical analysis for significance for crosses using pUASpGFP-M6FL-3′UTR with GAL4 drivers, CsprHS83 GFP-M6FL-3′UTR and myosin VI mutant. These crosses were done in parallel. The other lines were individually tested, so we did not include them in the statistical analysis. The different superscripts in the fertility column indicate significant difference (p<0.01).

### Immunofluorescence and electron microscopy and image acquisition

Testes were dissected and cysts were isolated as described previously [44]. F-actin was visualized with Alexa-568 or Alexa-633 labeled phalloidin (Invitrogen, Carlsbad, CA). Affinity-purified rabbit anti-Arp3 antibody or mouse anti-cortactin antibody (4F11, Millipore, Billerica, MA) were used and visualized using anti-rabbit or mouse secondary antibody conjugated to Alexa-568, respectively. Image acquisition was by confocal microscopy (TCS SP2; Leica, Wetzlar, Germany) using 488-, 561 and 633-nm lasers. For electron microscopy, isolated cysts were collected, decorated by S1 fragments, fixed, embedded, cut into sections, stained, and examined as previously described [3], [33].

### Determination of actin cone size and shape

Isolated cysts were stained with labeled phalloidin and visualized by confocal microscopy using a 63× lens. Images were captured of actin cones that had moved synchronously and traveled more than one third the length of the testis. Actin cones that were judged to lie horizontally on the slide were selected and their length, front width, and body width were measured using ImageJ software (NIH). Student's t tests were performed to evaluate the significance of differences in measurements between genotypes.

## Supporting Information

Figure S1 **Ultrastructure of actin cones is altered when myosin VI is mislocalized in mutant animals.** Examples of S1 decorated cones when GFP-HNPMD (Gtail deleted) transgene is expressed in myosin VI mutant background. (insets, i–iii), Small regions of actin cones at high magnification. Big arrow indicates the direction of cone movement and small arrows indicate areas near the cone center lacking actin filaments. mi, mitochondria; ax, axoneme. Bars, 1 µm.
(TIF)

Figure S2 **Ultrastructure of actin cones is altered when myosin VI is mislocalized in wild-type animals.** Examples of S1 decorated actin cones when GFP-HNPMD (Gtail deleted) transgene is expressed in a wild-type animal. Big arrow indicates the direction of cone movement and small arrows indicate asymmetry of cone front domains. mi, mitochondria; ax, axoneme. Bars, 1 µm.
(TIF)
