# Supplementary figures and images for: Myosin VI Regulates Actin Structure Specialization through Conserved Cargo-Binding Domain Sites
Source: PLoS One. 2011 Aug 11;6(8):e22755. doi: 10.1371/journal.pone.0022755 (PMC3154908; doi:10.1371/journal.pone.0022755)

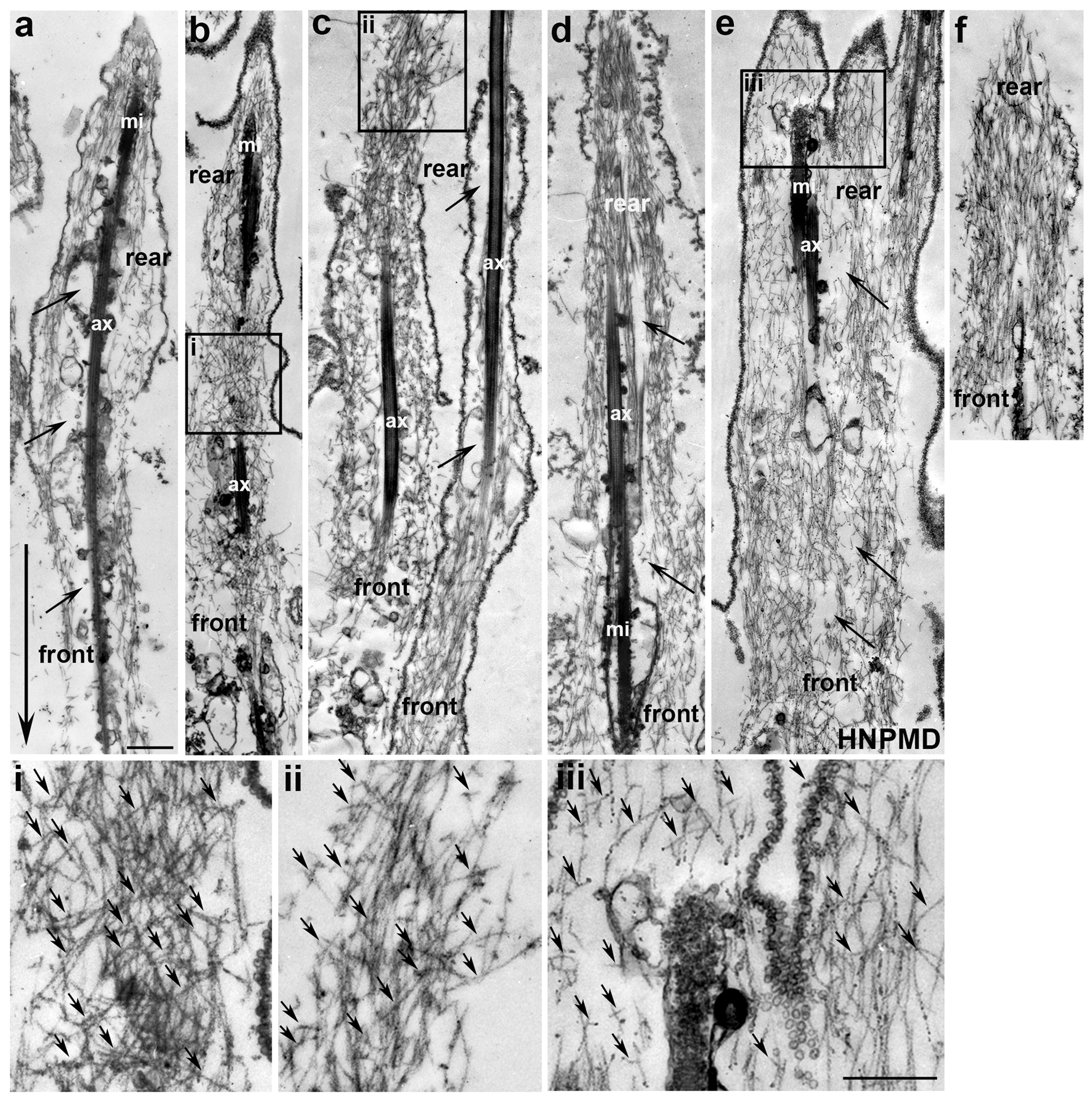

Supplement: Figure S1 — Ultrastructure of actin cones is altered when myosin VI is mislocalized in mutant animals. Examples of S1 decorated cones when GFP-HNPMD (Gtail deleted) transgene is expressed in myosin VI mutant background. (insets, i–iii), Small regions of actin cones at high magnification. Big arrow indicates the direction of cone movement and small arrows indicate areas near the cone center lacking actin filaments. mi, mitochondria; ax, axoneme. Bars, 1 µm. (TIF) [file pone.0022755.s001.tif]

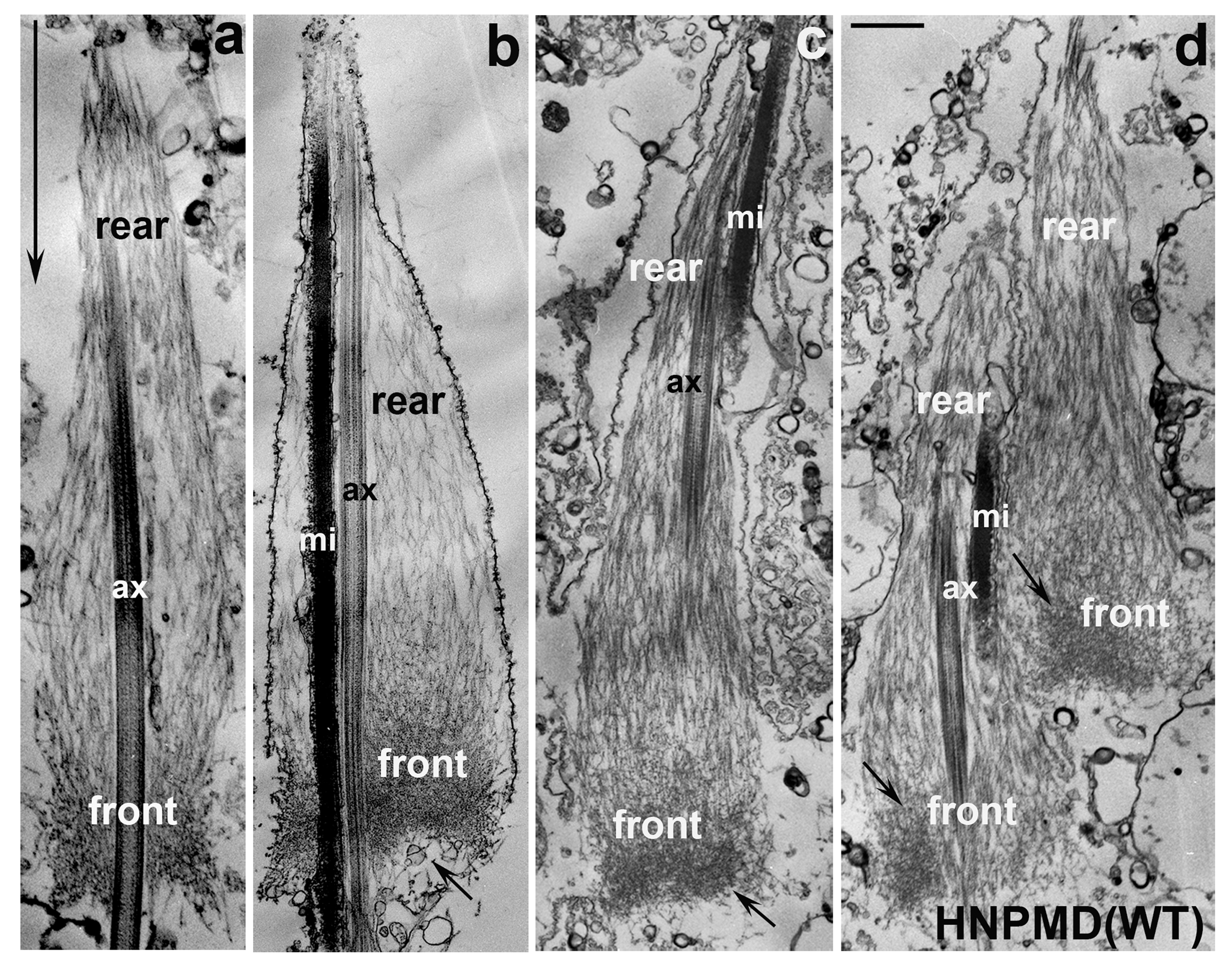

Supplement: Figure S2 — Ultrastructure of actin cones is altered when myosin VI is mislocalized in wild-type animals. Examples of S1 decorated actin cones when GFP-HNPMD (Gtail deleted) transgene is expressed in a wild-type animal. Big arrow indicates the direction of cone movement and small arrows indicate asymmetry of cone front domains. mi, mitochondria; ax, axoneme. Bars, 1 µm. (TIF) [file pone.0022755.s002.tif]
